# Structural Insight into DFMO Resistant Ornithine Decarboxylase from *Entamoeba histolytica*: An Inkling to Adaptive Evolution

**DOI:** 10.1371/journal.pone.0053397

**Published:** 2013-01-11

**Authors:** Preeti, Satya Tapas, Pravindra Kumar, Rentala Madhubala, Shailly Tomar

**Affiliations:** 1 Department of Biotechnology, Indian Institute of Technology Roorkee, Roorkee, Uttarakhand, India; 2 School of Life Sciences, Jawaharlal Nehru University, New Delhi, India; Technion-Israel Institute of Technology, Israel

## Abstract

**Background:**

Polyamine biosynthetic pathway is a validated therapeutic target for large number of infectious diseases including cancer, giardiasis and African sleeping sickness, etc. α-Difluoromethylornithine (DFMO), a potent drug used for the treatment of African sleeping sickness is an irreversible inhibitor of ornithine decarboxylase (ODC), the first rate limiting enzyme of polyamine biosynthesis. The enzyme ODC of *E. histolytica* (*Eh*ODC) has been reported to exhibit resistance towards DFMO.

**Methodology/Principal Finding:**

The basis for insensitivity towards DFMO was investigated by structural analysis of *Eh*ODC and conformational modifications at the active site. Here, we report cloning, purification and crystal structure determination of C-terminal truncated *Entamoeba histolytica* ornithine decarboxylase (*Eh*ODCΔ15). Structure was determined by molecular replacement method and refined to 2.8 Å resolution. The orthorhombic crystal exhibits *P*2_1_2_1_2_1_ symmetry with unit cell parameters *a* = 76.66, *b* = 119.28, *c* = 179.28 Å. Functional as well as evolutionary relations of *Eh*ODC with other ODC homologs were predicted on the basis of sequence analysis, phylogeny and structure.

**Conclusions/Significance:**

We determined the tetrameric crystal structure of *Eh*ODCΔ15, which exists as a dimer in solution. Insensitivity towards DFMO is due to substitution of key substrate binding residues in active site pocket. Additionally, a few more substitutions similar to antizyme inhibitor (AZI), a non-functional homologue of ODCs, were identified in the active site. Here, we establish the fact that *Eh*ODC sequence has conserved PLP binding residues; in contrast few substrate binding residues are mutated similar to AZI. Further sequence analysis and structural studies revealed that *Eh*ODC may represent as an evolutionary bridge between active decarboxylase and inactive AZI.

## Introduction

*Entamoeba histolytica* is responsible for causing amoebiasis, amoebic liver abscess and amoebic colitis in humans. It is the third major and a dangerous public health problem in the world [1], [2]. Though a small number of drugs including metronidazole, emetine, tinidazole, chloroquine and nitrazoxanide are used for the treatment of the disease, most of them are associated with numerous side effects. In some cases, frequent use of these drugs has led to the development of clinical drug resistance in the pathogen [3], [4]. Thus, it is crucial to identify and elucidate a potent metabolic pathway in *E. histolytica* which could be set as a therapeutic target for development of new anti-amoebic drugs.

In last few decades, the polyamine metabolic pathway in protozoan diseases including African sleeping sickness [5], giardiasis [6] and leishmaniasis [7] has emerged as a potential therapeutic target [8]. The polyamines such as putrescine, spermidine and spermine are essential polycationic compounds, which are involved in various cellular processes that govern cell growth and proliferation [9]. Subsequently, the actively proliferating cells have higher concentrations of polyamines. The intracellular concentrations of polyamines are tightly regulated by different mechanisms including biosynthesis, inter-conversion, degradation, and uptake from the surrounding through polyamine transporter. The failure in regulation of polyamine levels in cells has been linked to various cancers. Hence, polyamine metabolic pathway is also a potential target for cancer treatment [10], [11], [12]. Consequently, not only the polyamine biosynthetic pathway but also the key components of polyamine homeostasis are potential therapeutic targets [8]. The two enzymes of polyamine biosynthesis pathway, ornithine decarboxylase (ODC) and S-adenosylmethionine decarboxylase (SAMDC) are highly-regulated and have a very short half-life by which cells quickly alter the levels of polyamines [13].

Ornithine decarboxylase catalyzes the first and rate-limiting step of polyamine biosynthetic pathway. L-ornithine is decarboxylated by ODC enzyme in the presence of cofactor pyridoxal-5′-phosphate (PLP) to produce putrescine. The enzymatic activity of ODC is tightly regulated by a distinct mechanism in which polyamines induce the expression of a regulatory protein called antizyme (AZ) by +1 ribosomal frameshifting [14]. AZ inhibits ODC enzyme activity by binding and disrupting active ODC homodimers, and subsequently marks the enzyme for ubiquitin-independent degradation by the 26S proteasome [15],[16]. Additionally, AZ negatively regulates the uptake of polyamines by repressing polyamine transporter [17]. Thus, polyamine homeostasis is maintained in a cell through polyamines themselves *via* a negative feedback system, by governing the synthesis of AZ protein.

Furthermore, in mammals, the activity of antizyme is negatively regulated by a protein called antizyme inhibitor (AZI). AZI binds to antizyme and blocks the binding of antizyme to ODC which down regulates ODC degradation as well as leads to ODC activation. AZI has higher binding affinity for antizyme as compared to ODC which results in antizyme sequestration and elevation of ODC levels [18],[19],[20],[21],[22],[23]. Previously, it has been reported that AZI is homologous to ODC and the major residues involved in catalytic activity of ODC are conserved in AZI [24]. However, AZI does not possess enzymatic activity due to changes in the sequence that lead to protein inability to bind cofactor PLP along with the failure in decarboxylation activity [24],[25],[26].

In *E. histolytica*, ODC is the only enzyme of polyamine biosynthetic pathway that has been reported to exist in the organism [27]. The analysis of polyamine content shows that considerable amount of putrescine is present in *E. histolytica*. While, very low levels of spermidine and no spermine is detected supporting the absence of other genes of polyamine biosynthetic pathway in *E. histolytica* genome [28],[29]. Interestingly, the comparison of *Eh*ODC kinetic parameters with other well characterized ODCs indicates that it has low substrate affinity and catalytic efficiency [29]. Moreover, DFMO, a suicide substrate inhibitor of ODC is used for the treatment of African sleeping sickness, a protozoan disease caused by *Trypanosome brucei gambiense* [30],[31]. Interestingly, DFMO being an effective drug against *T. brucei gambiense* is reported to have relatively poor effect on the more virulent strain *T. brucei rhodesiense* [32],[33]. Furthermore, the ODC of *E. histolytica*, being a pathogenic strain from protozoa kingdom, is insensitive to DFMO due to sequence divergence in the substrate binding residues [29],[34],[35],[36]. Natural resistance to DFMO within the same *Trypanosome* species as well as within the protozoa kingdom draws attention towards the sequence and structural divergence for their evolutionary adaptation.

In this study, we have determined the crystal structure of *Eh*ODC to elucidate the structural features responsible for DFMO insensitivity and low substrate binding affinity. Furthermore, detailed comparative sequence and structural analysis was performed with functional ODCs and non-functional ODC homologue i.e. AZI to investigate the evolutionary status of *Eh*ODC.

## Materials and Methods

### Reagents

Restriction enzymes *Nde*I, *Xho*I, T4-DNA ligase and phusion polymerase were purchased from NEB. Primers were ordered from Integrated DNA Technology. HisTrap HP Ni Sepharose column and Hiload 16/60 Superdex 200 pg size exclusion column were obtained from GE healthcare. For crystallization, PEG ION screens were obtained from Hampton Research (Hampton Research Inc. Aliso Viejo, CA). The plasmid pET30a containing full length of *Eh*ODC was taken as template for sub-cloning [29].

### Cloning of C-terminal truncated *Eh*ODC

Polymerase Chain Reaction (PCR) amplification was carried out using forward primer 5′-ATATCCATATGAAACAAACATCTCTAGAAG-3′ and reverse primer 5′- GAACCTCGAGTCATTCAATTGACTTAGGGATTTGAAT-3′ with *Nde*I and *Xho*I restriction enzyme sites respectively to obtain DNA fragment encoding the C-terminal 15 residues truncated *Eh*ODC (*Eh*ODCΔ15). The previously cloned full-length *Eh*ODC was used as a template in the PCR reaction [29],[36]. PCR was performed in a 50 µl reaction mixture containing 10 µl of 5× HF phusion buffer supplied with the enzyme, 300 µM of dNTP mix, 6.25 pmol of each of forward and reverse primers, 10 ng of template DNA, 1 µl of 2.5 U/µl phusion polymerase and water. The reaction was performed with initial denaturation at 95°C for 30 s, followed by 30 PCR cycles of denaturation at 95°C for 30 s, annealing at 51°C for 60 s and extension at 72°C for 1 min and 15 s. A final extension was carried out at 72°C for 15 min. The resultant PCR product was subcloned into *Nde*I and *Xho*I sites of pET-28c with His_6_-tag preceding the N-terminal and tobacco etch virus (TEV) protease cleavage site to allow the removal of tag from recombinant protein. Ligated product was transformed into freshly prepared *E. coli* DH5α competent cells. Kanamycin resistant transformants were selected and grown in LB broth supplemented with 50 µg/ml kanamycin. The pET28-*Eh*ODCΔ15 plasmid was isolated and right size insert in the construct was confirmed by DNA sequencing from TCGA, New Delhi.

### Expression and purification

The pET28-*Eh*ODCΔ15 plasmid containing truncated *Eh*ODC gene was transformed into *E. coli* BL21 (DE3) competent cells. For protein expression, transformed BL21 (DE3) cells were grown at 37°C to an optical density of ∼0.6 at 600 nm (OD_600_) and induced with 0.5 mM isopropyl-ß-thiogalactopyranoside (IPTG). Induced cultures were transferred to 18°C and cells were grown for ∼14 h. Cells were harvested by centrifugation at 5,000 rpm at 4°C and cell pellets were stored at −20°C until further use. For protein purification, cell pellets from 1 litre culture were re-suspended in 20 ml of ice cold binding buffer containing 50 mM Tris HCl (pH 7.5), 40 mM imidazole, 250 mM sodium chloride, 2 mM phenylmethylsuphonyl fluoride (PMSF) and 5% glycerol (v/v). Lysozyme was added to a final concentration of 100 µg/ml and kept on rocking platform at 4°C for 45 min. Cells were disrupted by sonication on ice with 50% amplitude and a pulse of 20 sec on and 60 sec off for 15 min. The lysate was centrifuged at 18,000 rpm for 45 min at 4°C to separate supernatant from cell debris. The supernatant was loaded onto 5 ml HisTrap HP affinity column pre-equilibrated with the binding buffer. Protein was eluted by running a linear gradient of 40–1000 mM imidazole in 60 ml of buffer A [50 mM Tris HCl (pH 7.5), 1 M imidazole, 250 mM sodium chloride and 5% glycerol (v/v)] at a flow rate of 1 ml/min. Eluted fractions were analyzed on sodium dodecyl sulfate-polyacrylamide gel electrophoresis (SDS-PAGE) and fractions containing pure protein were pooled together. To remove the N-terminal His-tag, TEV protease was added to the sample with protein to TEV ratio 1∶20 and incubated for ∼12 h at 4°C and simultaneously dialyzed against buffer A without imidazole. To remove uncleaved His-tag protein and His-tag TEV protease, the sample was again loaded onto 5 ml HisTrap HP column. Flow-through containing *Eh*ODCΔ15 without His-tag was collected and concentrated using a 10 kDa cut-off Amicon Ultra-15 concentrator (Millipore, Bedford, Massachusetts, USA). The concentrated protein was loaded onto HiLoad 16/60 prep grade Superdex 200 size-exclusion chromatography column pre-equilibrated with buffer B containing 30 mM HEPES-Na (pH 7.5), 250 mM NaCl, 1 mM EDTA, 10% (v/v) glycerol and 1 mM DTT. The major peak fractions containing pure protein were pooled and concentrated to 5 mg/ml. Homogeneity of purified *Eh*ODCΔ15 protein was analysed on 12% SDS-PAGE. Protein concentration and yield were determined using the Bio-Rad protein assay kit with bovine serum albumin (BSA) as a standard.

### *Eh*ODCΔ15 enzymatic activity

To confirm that the truncation of 15 residues from C-terminus does not inactivate *Eh*ODC, ornithine decarboxylation activity of purified protein and production of putrescine was spectrophotometrically determined using the method developed by Badolo *et al* [37]. Enzymatic activity of the purified *Eh*ODCΔ15 protein was compared with full-length *Eh*ODC [36].

### Gel filtration analysis

The average molecular weight of *Eh*ODCΔ15 was determined using size exclusion chromatography and compared with previously characterized full-length *Eh*ODC [36]. In brief, the purified protein was concentrated to 5 mg/ml and was injected onto HiLoad 16/60 Superdex 200 gel filtration chromatography column using ÄKTA purification system (GE Healthcare). Protein was allowed to pass through the column at a rate of 0.5 ml/min. For the molecular weight estimation of *Eh*ODCΔ15, the elution profile of the major peak containing purified protein was compared with the elution profile of the standard Gel Filtration HMW Calibration kit molecular weight markers (GE healthcare).

### Crystallization

For crystallization, purified *Eh*ODCΔ15 protein was concentrated to 12.5 mg/ml in 30 mM HEPES-NaOH buffer (pH 7.5) containing 1 mM EDTA, 0.25 M NaCl, 1 mM DTT and 10% (v/v) glycerol. Crystallization trials were performed using the sitting drop vapour diffusion method in 96 well plates (Hampton Research) at 20°C and 4°C. The drops were prepared by mixing 2 µl of protein solution with 1 µl of reservoir solution and equilibrated against 80 µl reservoir solution. Hampton Research crystallization screens Crystal screen, Crystal screen 2 and PEG/ION screen (Hampton Research, USA) were used to explore the initial crystallization conditions. Crystals were obtained in PEG ION screen containing 20% PEG 3350 in 0.2 M LiCl solution maintained at pH 6.8. Diamond shaped crystals of *Eh*ODCΔ15 appeared in four months at 20°C. Prior to data collection, crystal was cryo-protected by bathing it in mother liquor containing 3% (v/v) ethylene glycol for 10 s. The crystal was flash-frozen under cryogenic conditions at 100 K using liquid nitrogen stream to prevent radiation damage during data collection.

### Data Collection and structure determination

The diffraction data of *Eh*ODCΔ15 were collected at 100 K using Cu Ka radiation generated by a Bruker Microstar-H rotating-anode generator assembled with MAR 345 imaging-plate system. The data were collected at 1.54 Å with a crystal-to-detector distance of 200 mm and 1° oscillation per image with 20 min exposure per frame. Crystal diffracted to 2.8 Å resolution. The data were indexed, integrated and scaled using HKL2000 program [38]. Table 1 summarizes data collection and processing statistics. The structure was solved by molecular replacement method using Molrep program of CCP4-6.0 suite [39]. The model was generated using previously reported crystal structure of human ODC (PDB ID: 2ON3) [40]. Non-crystallographic symmetry restraints were applied throughout the refinement stages using four *Eh*ODCΔ15 molecules in the asymmetric unit. Structure refinement was performed using CNS v.1.2, Phenix v 1.7.2-869, and REFMAC 5.2 refinement tools [41],[42],[43]. Rounds of model building were carried out using program Coot v 0.6.2 [44]. The quality of the model was evaluated by PROCHECK [45].

**Table 1:** Statistical representation of data collection and structure refinement parameters along with quality of the model accessed by Ramachandran plot.

*Data collection*	
Space group	*P*2_1_2_1_2_1_
*Unit cell parameters*	
*a* (Å), *b* (Å), *c* (Å)	76.66, 119.28, 179.28
Resolution (Å)	99.5–2.87 (2.92–2.87)^a^
Number of reflections	35570
Completeness (%)	92.1(59.0)^a^
Mean redundancy	3.4 (2.1)^a^
I/σ	4.82 (2.0)^a^
R_merge_ b (%)	0.150 (0.670)^a^
*Refinement*	
Resolution (Å)	99.5–2.87 (2.92–2.87)^a^
*Number of non-H atoms in asymmetric unit*	
Protein	10484
Water molecules	101
*R*-factor (%)	25.3
*R*_free_ c value (%)	29.9
Average *B*-factor (Å^2^)	54.4
*Rms deviations*	
bond lengths (Å)	0.005
bond angles (°)	0.831
*Ramachandran plot*	
Residues in favored region (%)	88.7
Residues in allowed region (%)	10
Residues in generously allowed region (%)	0.9
Residues in outlier region (%)	0.4

a value in parentheses are for the highest resolution shell.

b R_merge_ = Σ | *I*−*Ī* |/Σ *I* | where I = observed intensity and Ī = average intensity.

c R_free_ = Σ (|F|_obs_−|F|_calc_|)/Σ |F|_obs_ where |F|_obs_ are observed structure factor amplitudes for a given reflection and |F|_calc_ are calculated structure factor amplitude.

### Sequence Analysis of *Eh*ODC

The sequence of *Eh*ODC, along with other functional ODCs and AZI were retrieved from NCBI database [46]. Multiple sequence alignment and phylogenetic tree of these sequences were obtained using ClustalW [47] for evolutionary variation analysis.

### Model generation for active site analysis

In the crystal structure of *Eh*ODCΔ15, the flexible loops missing in one subunit were present in the other subunits. Therefore, coordinates for missing loops near active site in the structure were generated by MODELLER 9.10 [48] using the solved crystal structure of one subunit of *Eh*ODC as a template. Evaluation of the steriochemical properties of obtained structure having built-in loops was performed using PROCHECK [45]. All the figures of structure and active sites were generated using PyMol [49].

## Results and Discussion

### C-terminal Truncation and Purification of *Eh*ODC

**Figure 1:**
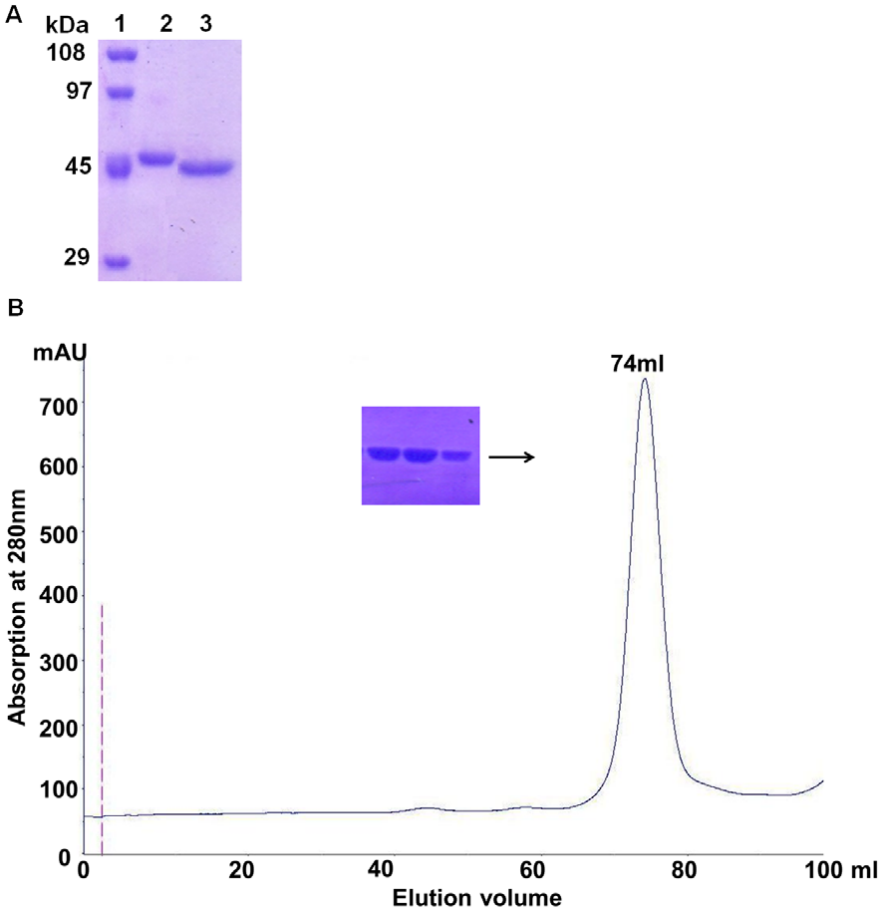
Purification and gel filtration profile of *Eh*ODCΔ15. **A**) 12% SDS-PAGE gel showing the affinity purified protein **Lane 1:** Molecular weight markers shown in kDa. **Lane 2–3:** Protein purified by affinity chromatography. **B**) Elution profile of the *Eh*ODC1Δ15 protein. The protein was eluted at a volume of 74 ml corresponding to molecule weight of ∼87 kDa. Insert shows the purified protein in 12% SDS-PAGE after gel filtration chromatography.

The ODC enzyme from *E. histolytica* belongs to fold type III group IV decarboxylase of a B_6_-dependent family, having eukaryotic ornithine decarboxylase characteristics [50],[51]. Under this classification, crystal structures are only available from three different sources including human, mouse and *Trypanosome brucei* ODC [52],[53],[54]. For crystal structure determination of *Eh*ODC, full-length protein was purified using the previously established protocol [36] and was used for crystallization experiments. However, extensive crystallization trials of full-length *Eh*ODC were unsuccessful. In order to decrease the conformational heterogeneity, it is a common practice to truncate the flexible N and/or C-terminal residues to facilitate the crystallization process. Therefore, *Eh*ODC sequence was examined to identify disordered regions using bioinformatics tools DisEMBL and GlobPlot [55],[56]. These programs predicted a fragment of approximately 13–17 residues at the C-terminus of *Eh*ODC to be flexible. Additionally, it has been reported that the truncation of 37 residues from the C-terminus of mouse ODC resulted in protein stability and has been crystallized successfully for structure determination [53],[57],[58]. The C-terminal sequence of *Eh*ODC shows similarity with mouse ODC in having a PEST like sequence [36]. Based on these observations, 15 residues were deleted from the C-terminus of *Eh*ODC. Expression and solubility of *Eh*ODCΔ15 construct was optimized by varying induction temperature (37°C, 25°C, and 18°C). Maximum solubility was observed at 18°C when induced with 0.5 mM IPTG for ∼14 h. Recombinant *Eh*ODCΔ15 was purified in three sequential purification steps, with yield of ∼5 mg per liter of *E. coli* culture. Elution profiles from the gel filtration column demonstrated that *Eh*ODCΔ15 exists in the dimeric form similar to full-length *Eh*ODC [36]. The purified protein exhibited a single band of approximately ∼45 kDa in 12% SDS-PAGE gel (Figure 1). The enzymatic activities of *Eh*ODCΔ15 and wild-type proteins were compared using previously established protocol [36]. The comparative analysis of both the full-length and *Eh*ODCΔ15 forms didn't show any notable difference in the activity indicating that the truncation of 15 residues from the C-terminus of *Eh*ODC does not affect its activity.

### Crystal packing

Crystallization of purified *Eh*ODCΔ15 was performed using sitting drop vapor diffusion method. Crystals were obtained at 20°C in Hampton PEG ion screen 4 containing 20% (v/v) PEG 3350, 0.2 M LiCl maintained at pH 6.8. Crystals belonged to the orthorhombic space group exhibiting *P*2_1_2_1_2_1_ symmetry with unit cell parameters *a* = 76.66, *b* = 119.28, *c* = 179.28 Å and *α* = *β* = *γ* = 90°. The crystal diffracted to 2.8 Å resolution, possessing four molecules per asymmetric unit and the solvent content was calculated to be 46.69% with a Matthews coefficient of 2.2 Å^3^ Da^−1^. Quality of the obtained structure was assessed with the PROCHECK program showing 88.7% of the residues in the favored region, whereas 10% in allowed, 0.9% in generously allowed and only 0.4% residues are observed in the disallowed region of Ramachandran Plot (Table 1).

**Figure 2:**
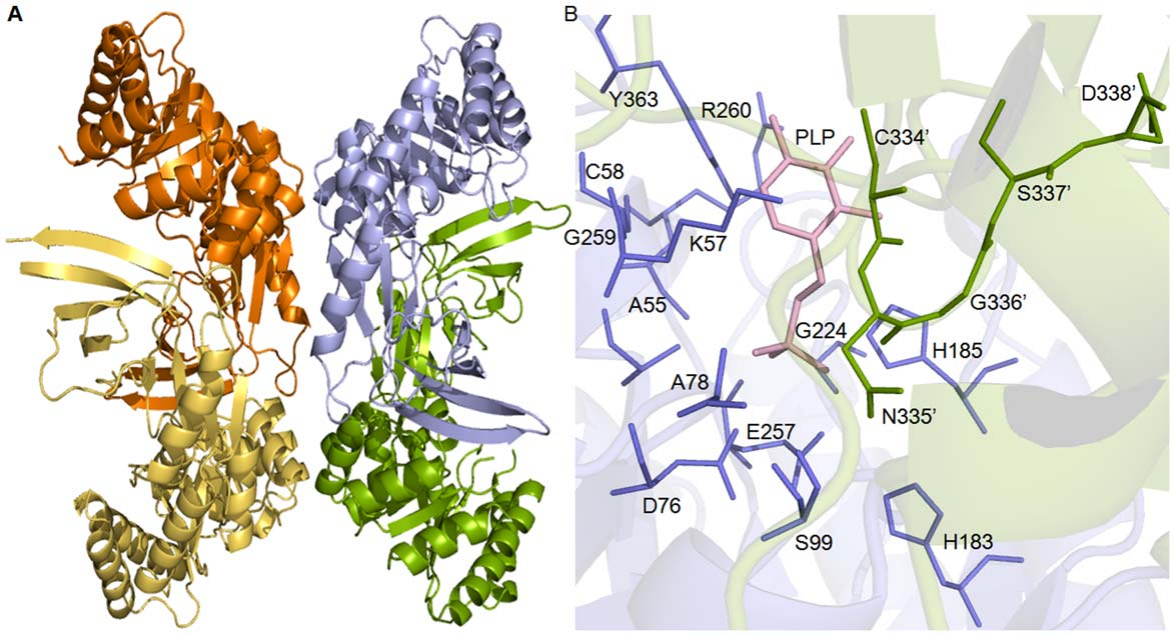
Schematic representation of overall structure of the model obtained after molecular replacement. **A**) Cartoon diagram of tetrameric model of *Eh*ODC showing AB-CD, dimer-dimer interface; **B**) Active site of *Eh*ODC at the interface of dimer where (′) denotes the residues from the other subunit.

The four monomers in the asymmetric unit of crystal are arranged as two separate dimers (subunits A, B and subunits C, D) facing each other at the convex surfaces. Each monomer in a dimer makes side to side contacts with each other forming an overall bent structure. Further, as the loops in a dimer interface are disordered and clear density was not observed, the central part of dimer forms a hollow structure. In the dimer, chain A and chain B are arranged in head to tail manner at origin (0,0,0) of orthorhombic unit cell (Figure 2). The β/α barrel of chain A and β sheet of chain B pose at origin and their counterpart extends along X-direction. Dimer of AB is situated along with X-axis by an angle of 30° approximately; whereas other dimer CD is situated at rotation angle of 180° with a screw distance of 19.1 Å that occupies approximately one quarter of unit cell. The crystallized structure of *Eh*ODCΔ15 consists of a tetramer. The asymmetric unit contains two dimers comprising of chain A, B, C, D. The total area of the molecule of *Eh*ODC containing four molecules was estimated to be 61227.6 Å^2^. Each dimer interacts with its symmetry mate to form dimer-dimer interfaces as A–B dimer interacts with C–D dimer (Figure 2). Interface area evaluated by PISA web server was averaged to 1373.4 Å^2^ which was 1599.8 Å^2^ and 1147.0 Å^2^ between B, A and D, C respectively [59].

**Figure 3:**
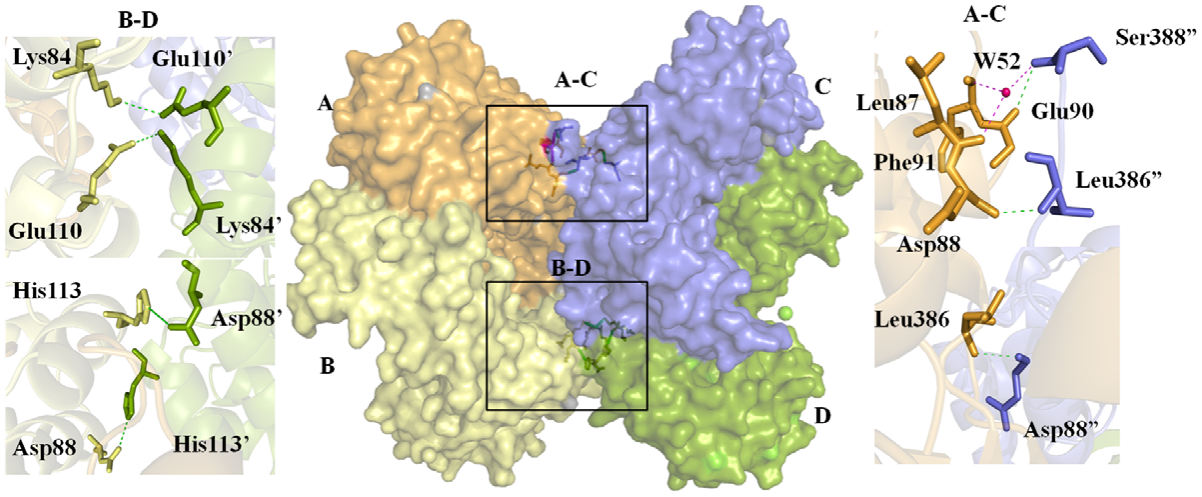
Tetrameric structure with dimer-dimer interaction. **A–C**) shows the interaction between chain A and chain C. **B–D**) indicates the interaction between chain B and chain D. Pink dashes shows the interaction of residues through water molecule and green dashes indicates the polar interactions. Symbol (″) and (′) denotes the residues of chain C and chain D, respectively.

In the tetrameric structure, residues Phe91 and Leu87 of chain A interact with Ser388 of chain C through a water molecule. In addition, Glu90 of chain A interacts to Ser388 of chain C through polar interaction. Similarly, Asp88 of chain A is forming direct interaction with residue Leu386 of chain C and *vice versa*. Residues Glu110 and His113 of chain B are at a distance of 3.1 Å and 3.3 Å from Lys84 and Asp88 of chain D showing polar interactions and *vice versa* (Figure 3).

### Overall structure and folding

**Figure 4:**
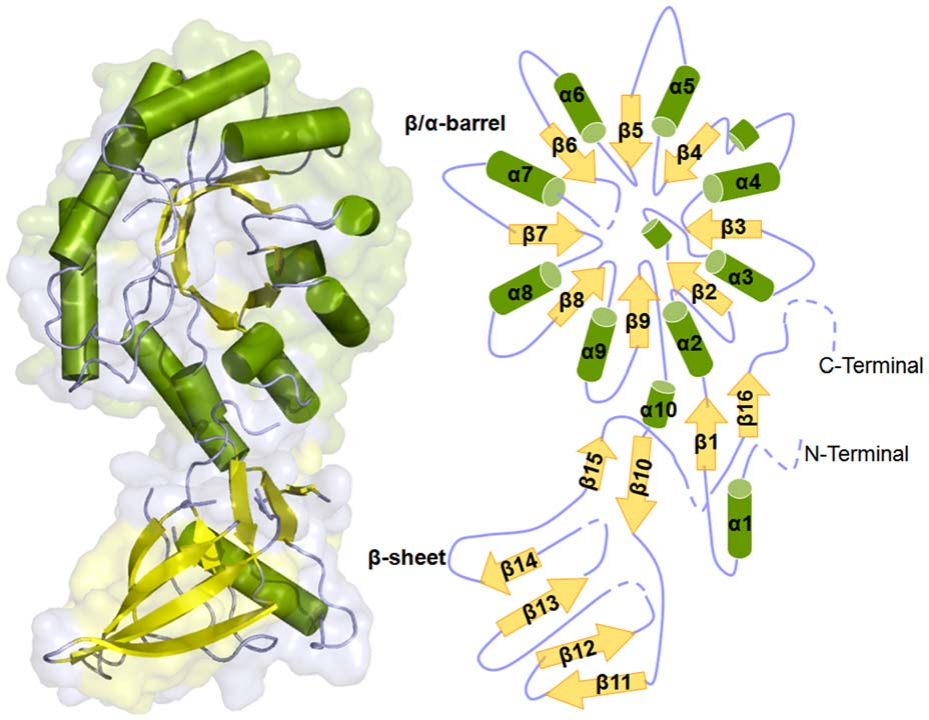
Crystal structure of *Eh*ODC monomeric subunit. **A**) Cartoon diagram of the monomer showing arrangement of barrel and sheet domain. **B**) Topology diagram of monomer of *Eh*ODC where helices are represented with cylinder and sheets with the arrows connected with loops, dashed line indicates the sequence missing in the structure.

Each monomer consists of β/α barrel and β-sheet domain which are arranged identical to previously known ODC structures (Figure 4). However, the tetramer arrangement displays a number of unusual features. Residues from barrel involved in contact and dimer formation are located at the surface or in proximity to sheet domain of opposite monomer. Interface residues of helices α5, α7, α8 and α9 of chain A barrel form extensive contacts with sheet domain S2 of chain B. All four chains in asymmetric unit showed similar structures and are involved in similar interactions. The analysis of dimer-dimer interactions exhibited large intermolecular distances of ∼4.0 Å. In a monomer, helix α1 is connected to sheet β1 (Gly27-Phe31) through a loop and enters the barrel. The barrel is composed of eight helices i.e. α2 (T33-N46), α3 (P62-L71), α4 (L80-L89), α5 (Y105-L114), α6 (I124-Y133), α7 (D163-K175), α8 (E194-F213) and α9 (F232-L246) followed by eight alternate β-strands β2 (R51-A55), β3 (G74-C77), β4 (I96-Y98), β5 (H118-V121), β6 (G138-R142), β7 (V182-F184), β8 (L219-D221) and β9 (R253-A256). The sheet domain comprises of eight randomly arranged β-strands which can be further divided into S1 and S2 β-sheets that are perpendicular to each other. Sheet S1 consisted of four sheets β10 (F267-S271), β15 (L355-F357), β16 (I381-T383) in addition to β1, which are roughly perpendicular to S2 containing β11 (H274-Q281), β12 (K284-S291), β13 (Y325-Y330) and β14 (A341-L345) (Figure 4). However, both domains are connected by two loops in between β1-α2 and α10-β10. The barrel and β-sheet domains of the monomeric subunits are associated in head to tail manner in the dimer. In addition, various polar interactions at the dimer interface including salt bridges and hydrophobic interactions are involved in the formation of dimer. The structure of *Eh*ODC has several highly mobile loop moieties that are depicted by dashed lines in Figure 4.

### Comparative analysis of active site architecture

**Figure 5:**
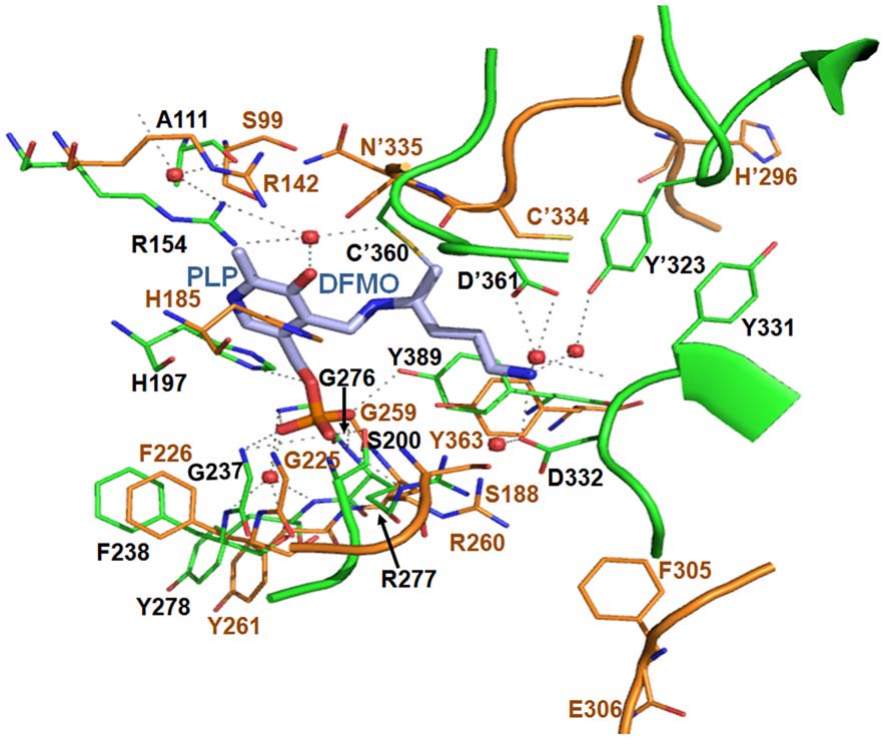
Superimposition of active site of *Eh*ODC with *Tb*ODC bound to DFMO. Residues of active site at the dimer interface are represented in sticks. *Tb*ODC residues are colored with green, *Eh*ODC residues are colored with orange. PLP and DFMO are colored with blue and polar interactions were indicated by black dashes; water molecule in shown in red sphere. Residues with (′) symbol are of opposite monomer.

The ODC enzyme is an obligate homodimer with two symmetry-related active sites located at the dimer interface. According to our previous report, *Eh*ODC enzyme is functionally active in the dimeric form [36]. As expected, the crystal structure of *Eh*ODC contains two equivalent active site pockets at the dimer interface formed by residues that are contributed from both the subunits (Figure 2). The proper orientation of active site formed by two subunits is highly essential for functionality of the enzyme. The active site in *Eh*ODC is mainly contributed by loops from both the subunits. For comparative analysis of active site, we superimposed the *Eh*ODC structure over *Tb*ODC complexed with DFMO. The super-imposition of monomers shows root mean square deviation (rmsd) of 1.18 Å whereas super-imposition of dimer shows rmsd of 1.7 Å. Active site superimposition of *Tb*ODC and *Eh*ODC shows that most of the conserved residues in active site of *Eh*ODC share same positions as in *Tb*ODC, however few residues pose in different orientation (Figure 5). His185, Gly259, Arg260 and Tyr363, the well conserved PLP binding residues of *Eh*ODC share the position and have orientation similar to the PLP binding residues (His197, Gly276, Arg277 and Tyr389 respectively) of *Tb*ODC (54, PDB ID: 2TOD). The side chains of these conserved residues interacting with PLP through polar interactions in *Tb*ODC are also expected to bind PLP and correctly orientation it into the active site of *Eh*ODC. Interestingly, Ser200 of *Tb*ODC is present in a loop and is seen interacting with PLP in *Tb*ODC structure (54, PDB ID: 2TOD). However, this residue (Ser188) is also conserved in *Eh*ODC, but shows small displacement from its expected position and has opposite orientation in the crystal structure of *Eh*ODC. Though, at this point it cannot be ruled out that the flexible loop of *Eh*ODC possessing Ser188 may approach the active site and may orient Ser188 in favourable position in the presence of PLP, whereas apo-enzyme might not restrict its position. Addition to this, Gly225 of *Eh*ODC shares exact position of Gly237 of *Tb*ODC, whose backbone carbon chain contributes to polar interactions. Apart from this, few residues interact with PLP through water molecules which include residues Phe238, Tyr278, Arg154 and Ala111 in *Tb*ODC. These residues are present at the same position in *Eh*ODC active site except Ala111 where it is substituted by Ser99 in *Eh*ODC. Overall, the architecture of *Eh*ODC for binding to PLP is similar to that of *Tb*ODC.

DFMO, a substrate analogue makes a stable covalent bond with conserved Cys residue in the active site of ODC enzyme and inhibits its catalytic reaction. Binding of DFMO in the proper orientation for covalent bond formation with its active site is also supported by its interaction with other residues that are there in the substrate binding pocket. To extricate the intricate structural details of *Eh*ODC responsible for low substrate affinity and/or DMFO insensitivity, structural comparison of *Eh*ODC active site architecture for substrate/DFMO binding was done with the active site of DFMO bound *Tb*ODC crystal structure (Figure 5) (54, PDB ID: 2TOD). In *Tb*ODC, Cys'360 from counterpart subunit plays the most critical role in DFMO binding by making a permanent covalent bond with the enzyme. However, covalent bond formation of Cys'360 with substrate L-ornithine has not been reported for any ODC enzyme. In addition to this, the next residue of *Tb*ODC Asp'361 helps to position the Cys'360 residue in proper orientation and also interacts with DFMO through a water molecule. In contrast, Cys'334 the conserved residue of *Eh*ODC that is expected to form a covalent bond with DFMO is slightly displaced from its position and has distinct orientation that is structurally unfavourable for covalent linkage with DFMO. In addition, the residue Asp'361 of *Tb*ODC is substituted by Asn'335 in *Eh*ODC, which is not expected to interact with DFMO. Furthermore, alpha-carbon backbone consisting residues Tyr331 and Asp332 in *Tb*ODC shows direct interactions with bound DFMO and these interactions play a role in proper DFMO molecule orientation in the active site pocket. However, these residues are mutated to Phe305 and Glu306 respectively in *Eh*ODC. Also, *Eh*ODC crystal structure reveals that the loop consisting of Phe305 and Glu306 residues is not located close to the active site thus may not contribute to DFMO binding. Moreover, residue Tyr'323 from other subunit of *Tb*ODC also supports the favourable orientation of DFMO by side chain hydroxyl group interaction with DFMO through a water molecule. Tyr'323 is replaced with His'296 in *Eh*ODC and the loop containing His'296 residue is positioned away from the active site (Figure 5). In contrast, the cofactor PLP and substrate L-ornithine are accommodated in *Eh*ODC active site with polar interactions to facilitate the catalysis (Figure 2) [29],[35]. These structural details indicate that amino acid substitutions in the active site of *Eh*ODC create a novel architecture which not only makes it resistant to DFMO but also lowers its catalytic efficiency by weakening substrate binding, as the reported Km values for L-ornithine for active but DFMO sensitive *T. brucei* and mouse ODC enzymes are 0.24 mM and 0.09 mM respectively, whereas for DFMO resistant *Eh*ODC it is 1.5 mM [29],[60]. Not only substituted residues, but also the displacement of loops (His'296 loop/Phe305 and Glu306 loop) away from *Eh*ODC active site seems to contribute towards DFMO insensitivity.

### *Eh*ODC sequence and structural comparison with ODC homologs

**Figure 6:**
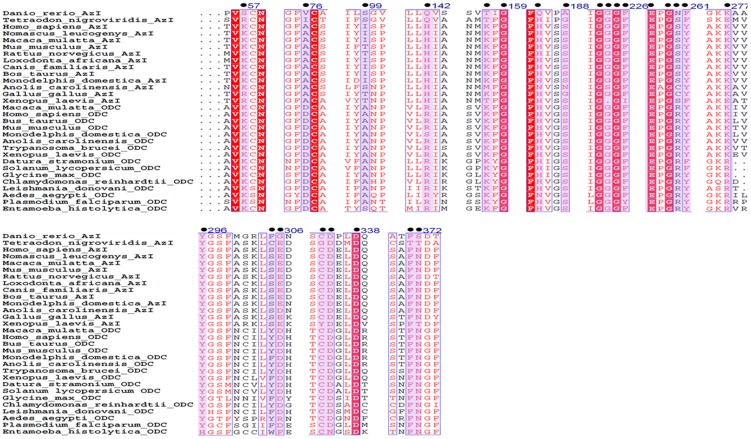
Multiple sequence alignment of ornithine decarboxylase and its homologues antizyme inhibitor to determine the conservation of sequence and mutation of active site and substrate binding residues. Circles indicate the residues important for enzymatic activity. Numbering is according to *Eh*ODC.

The novel active site architecture revealed from the crystal structure of *Eh*ODC and previously reported low catalytic efficiency of the enzyme hints towards possible adaptive evolution which lead to DFMO insensitive. AZI is an inactive ODC homolog that possesses a broader active site due to unusual packing of AZI dimers [61]. The architecture of AZI active site does not favour the accommodation of substrate as well as the co-factor for enzyme catalysis, which makes it an inactive homolog of ODC. Recently, it has been proposed that several homologs of ODCs including putative antizyme inhibitors apparently arise independently through evolution [46]. Robust sequence analysis and active site structure comparisons were performed to explore the evolutionary relationship of *Eh*ODC with respect to ODC homologs including AZI and to uncover the possibility of additional *Eh*ODC functions. Multiple sequence alignment was done and phylogenetic tree was generated for ODC homologues including functional ODC and nonfunctional AZI (Figure 6).

**Figure 7:**
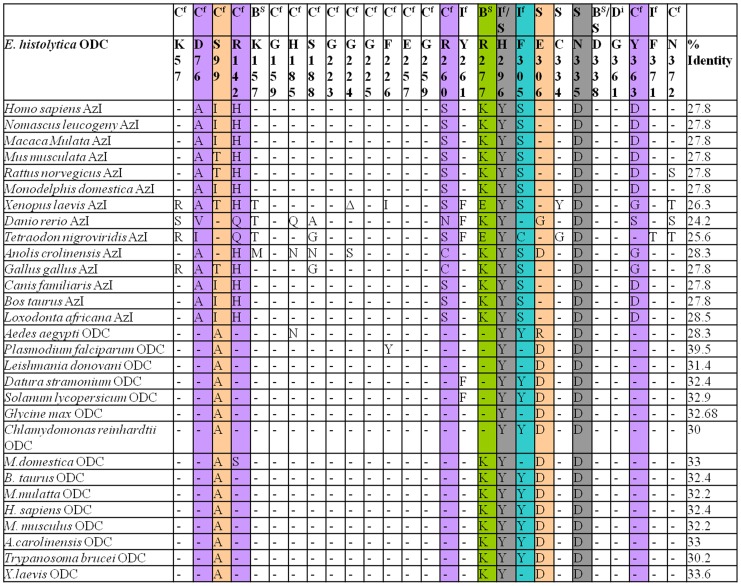
Sequence analysis of ODC and antizyme inhibitor, comparing the active site residues of ODC/AZI from various organisms. Abbreviation denoted: C^f^ for cofactor binding; B^s^ salt bridge formation; S substrate binding residues; I^f^ dimer interface residues; D^i^ important for dimer formation. Species with the name of protein are shown on left side. **Colour indication:** Violet columns signifies the mutation in AZI; Orange columns signifies the mutated residues in *E. histolytica* ODC which are similar to AZI; Gray shows the unique mutations in *Eh*ODC which is neither conserved in ODC nor in AZI; Blue indicate the mutation in *Eh*ODC which are rarely found in AZI and functional ODC; Olive color point out the mutations in *Eh*ODC which are similar to some ODC. Sequence analysis and numbering has been done according to *Eh*ODC. Residues which are not conserved are shown by single letter, the conserved residues are indicted by – and Δ indicates the deleted amino acids. % identity indicates the identity of *Eh*ODC sequence with other homologous ODC sequences [46].

AZI is an inactive homolog of ODC which has lost decarboxylation activity due to mutation of critical residues in the active site [24],[25],[26],[46],[62]. However, they are important in mammals as they are responsible for antizyme down regulations, thus regulate the ODC activity in cell system [21]. In this study, we have identified 27 residues from sequence alignment of ODCs from different organisms responsible for the formation of active site pocket and in ODC enzyme dimerization (Figure 7). Out of 27 residues, 16 residues contribute to the active site formation by interacting with cofactor PLP, 5 residues for substrate binding, 3 residues for salt bridge formation, 3 residues as critical interface residues and 1 residue for dimerization (Figure 7).

**Figure 8:**
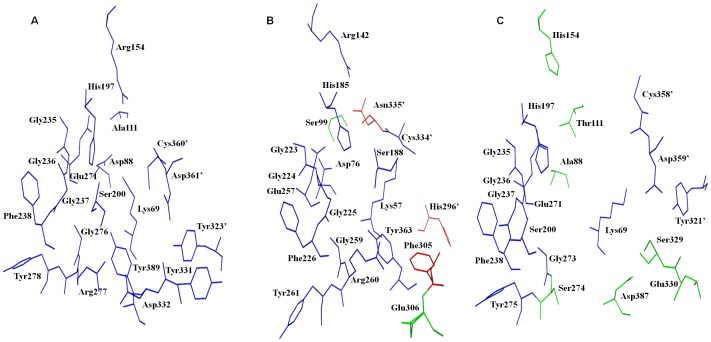
Active sites comparison of functional ODC, antizyme inhibitor and *Eh*ODC. **A**) Human ODC active site residues colored in blue. **B**) *Eh*ODC active site residues identical to human ODC colored blue, residues identical to AZI colored green and unique to *Eh*ODC colored red. **C**) AZI interface region showing residues identical to human ODC in blue and those are mutated colored green.

From various mutational studies, it is reported that a conserved Lys (Lys57 in *Eh*ODC) is important as it forms Schiff base with PLP which is later displaced by L-ornithine that undergoes decarboxylation through nucleophilic attack via a conserved Cys (Cys334′ in *Eh*ODC) [36],[63],[64],[65]. However, both the residues are well conserved in both functional ODCs and AZI (except in few AZIs). In all functional ODCs including ODCs from *T. brucei*, *Homo sapiens* (*Hs*ODC) and mouse, residue Ala111 and Arg154 (*Hs*ODC) are highly conserved and interact with PLP through water. Interestingly, in *Eh*ODC, though Arg142 is conserved, however Ala111 is uniquely substituted with Ser99. AZI possesses substitution at both the positions with Ala to Thr/Ile/Ser and Arg to His/Gln that make AZI incapable of binding to PLP. Out of sixteen PLP binding residues, AZIs have major mutations in five positions whereas *Eh*ODC possesses a single mutation at position 99 with substitution of Ala to Ser (Figure 7 and Figure 8).

In *Hs*ODC, five residues Tyr323, Tyr331, Asp332, Cys360 and Asp361 are reported to be key active site residues which interact with L-ornithine and these residues are highly conserved in all functional ODCs (Figure 7). However, *Eh*ODC is an exception where only Cys334 is conserved while both Tyr and both Asp residues are substituted by His296, Phe305, Glu306 and Asn335 respectively. The substitution of active site residues at His296 and Asn335 positions are unique to *Eh*ODC as these two residues are found to be conserved as Tyr323 and Asp361 in both AZI as well as in functional ODC. Interestingly, substitution at Glu306 instead of Asp332 is similar to AZIs, the inactive homologs of ODCs. However, in AZIs only Asp332 is substituted by Glu, whereas other four residues are mostly conserved (Figure 7).

Furthermore, in case of *Tb*ODC and other functional ODCs, Tyr331 contributes to form an aromatic zipper responsible for complementary packing in two monomers [54],[54]. In AZI, this is mutated to Ser rendering a loose contact between monomers [61]. But in *Eh*ODC, same residue is substituted by aromatic amino acid Phe305 that is expected to perform same job in aromatic zipper. The mutation of Tyr to Phe is also reported in *Plasmodium falciparum*, *Leishmania donovani* and *Glycine max* ODCs (Figure 7, Figure 7) [46],[66].

AZI genes have accumulated mutations in key residues that are important for ODC activity. In *Petromyzon marinus*, the homologue of AZI as classified on the basis of conserved key amino acid residues was found to be a functional ODC [67]. In contrast to this, ODC from *Aedes aegypti* is found to be enzymatically non-functional [46]. Thus, mutations in and around active site, ranging from substitution of one residue to substitution of fourteen residues in single polypeptide may cause enzyme inactivation. In *T. nigroviridis*, 11 residues are altered in the active site whereas in mammals 4 residues are altered to convert a functional ODC to a nonfunctional homolog [46]. However, in *Drosophila melanogaster*, though all 18 key residues of active site are conserved, but a single mutation of Asp332Tyr hinders dimer formation in ODC in addition to cofactor and substrate binding, which makes it a nonfunctional ODC.

**Figure 9:**
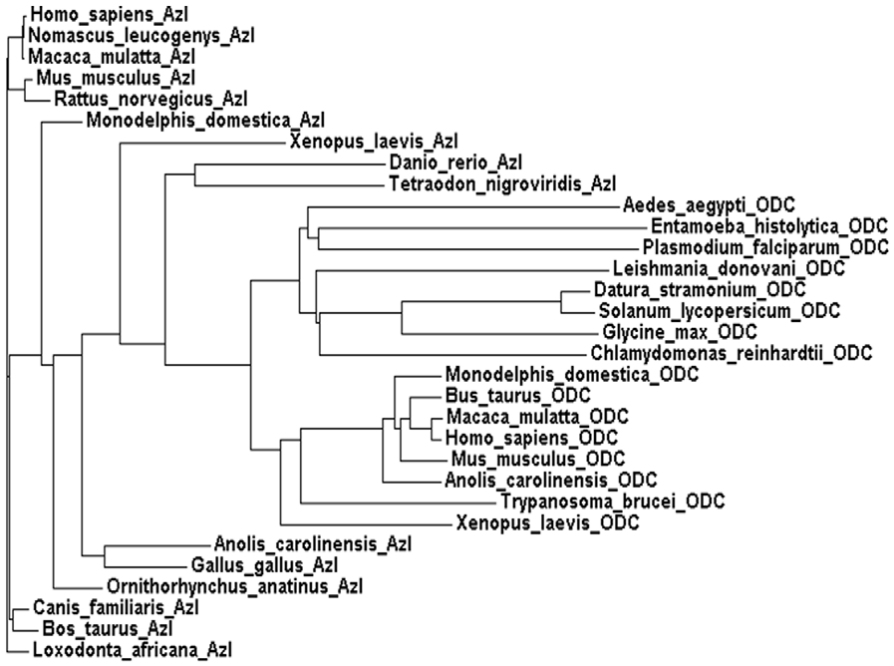
Phylogenetic relationship of *Eh*ODC with antizyme inhibitor and ODC. Sequence of ODC and their evolutionary related homologous were retrieved from various sources. **Antizyme inhibitor** of *Homo sapiens* (BAA23593.1), *Nomascus leucogenys* (XP_003256127.1), *Macaca mulatta* (XP_002805501.1), *Mus musculus* (AAB87464.1), *Rattus norvegicus* (BAA23594.1), *Monodelphis domestica* (XP_001369332.1), *Xenopus laevis* (NP_001087584.1), *Danio rerio* (BAB84695.1), *Tetraodon nigroviridis* (ENSTNIT00000008148.1), *Anolis carolinensis* (XP_003219500.1), *Gallus gallus* (NP_001008729.1), *Ornithorhynchus anatinus* (XP_001506230.1), *Canis familiaris* (XP_849306.1), *Bos Taurus* (NP_001076080.1), *Loxodonta africana* (XP_003408472.1). **Ornithine decarboxylase sequence from** *Aedes aegypti* (EAT48998.1), *Entamoeba histolytica* (AAX35675.1), *Plasmodium falciparum* (AAF14518.1), *Leishmania donovani* (AAA29259.1), *Datura stramonium* (CAA61121.1), *Solanum lycopersicum* (NP_001234616.1), *Glycine max* (CAD91349.1), *Chlamydomonas reinhardtii* (CAE46409.1), *Monodelphis domestica* (XP_001371947.1), *Bos Taurus* (AAA92339.1), *Macaca mulatta* (NP_001185615.1), *Homo sapiens* (NP_002530.1), *Mus musculus* (NP_038642.2), *Anolis carolinensis* (XP_003215471.1), *Trypanosoma brucei* (AAA30219.1), *Xenopus laevis* (CAA39760.1).

The evolutionary relationship of ODC and AZI can be evaluated by considering the root of phylogenic tree which connects the branch of both homologs. Evidences indicated that both the homologs are from same subfamily and have evolved and diverged according to their function. In phylogenetic tree, the group of AZI and ODC make different clusters according to the sequence alignment. Interestingly, *Eh*ODC is clustering to the ODC group just beneath the *Aedes aegypti* which is a nonfunctional ODC due to His197Asn and Asp332Arg substitutions as shown in Figure 7 and Figure 9. ODC of *Aedes aezypti*, being a non-functional ODC, represents the border line of functional ODCs and nonfunctional AZI. *Eh*ODC, the enzyme with low catalytic efficiency is found to be more evolutionarily related to nonfunctional ODC of *Aedes aezypti* and AZIs. These evidences from sequence alignment and phylogeny profile of *Eh*ODC allow us to establish the fact that during the course of evolution it gained DFMO resistance by acquiring critical alternation in its sequence similar to both functional ODCs and non-functional AZI. Though, the evolutionary changes in the sequence also influenced its catalytic efficiency. However, the possibility of additional biological role of *Eh*ODC such as antizyme inhibitory activity needs to be investigated.

### Conclusion

In the present report, we successfully determined the 3D structure of *Eh*ODC to elucidate its intricate active site architecture that made it DFMO insensitive. Further on the basis of sequence analysis, we unveiled many unique characteristics of *Eh*ODC that show similarity with both functional ODC and non-functional AZI. *Eh*ODC exists as a dimer like other functional ODCs and in contrast AZI is monomer in solution due to weaker interaction between two monomers. Interface of *Eh*ODC shows 45 contacts and 16 hydrogen bonds in addition to salt bridges which stabilize the dimer. As studied in AZI structure (mouse AZI), only 43 contacts and 15 hydrogen bonds are reported which lack salt bridge formation and make interface less interactive as compared to ODC [61]. Structure of *Eh*ODC at 2.8 Å revealed two salt bridges between Lys157-Asp238 at a distance 2.9 Å and Asp122-Arg277 at 3.2 Å. Same salt bridges are also reported in *Hs*ODC that contributes to dimerization. Though, same residues i.e. Lys169, Asp364, Asp134, and Lys294 are conserved in AZI (mouse), still residues do not approach to form the salt bridge [61]. Furthermore, AZI is inefficient to bind to PLP consequently unable to carry out decarboxylation reaction. The structure of AZI (mouse) reveals that the active site is too wide to make suitable pocket for substrate and PLP binding. However, *Eh*ODC binds to PLP and catalyzes decarboxylation of L-ornithine and relatively less active as compared to other active ODCs. It is interesting to note that though *Eh*ODC possesses similar property with other ODC on the basis of structure and function, it shares some similarity with AZI based on amino acid sequence. Firstly, the substrate binding residue Asp332 (*Hs*ODC) is conserved in functional ODCs where in *Eh*ODC same residue is altered to Glu306 and Glu is well conserved in AZI. Secondly, PLP binding residue Ala is altered to Ser in *Eh*ODC and such alternation is reported in AZI of *Danio rerio, Tetraodon nigroviridis* and *Anolis crolinensis*. Thirdly, *Eh*ODC possesses unique mutations at His296 and Asn335 those are neither reported in any functional ODC or AZI. Such alternation of critical residues particularly in protozoa provides the evidences of adaptive evolution of ODC. AZI dependent ODC regulation is only reported in higher organisms and absent from lower organisms. Even such regulation is not reported in protozoa till date. However, it can be hypothesized that ODC in protozoa takes the modification towards AZI though it functions less efficently as an active ODC and its function as AZI needs to be investigated.

Our study will facilitate to investigate the molecular evolution of ODCs and AZI. It also suggests additional functional properties for *Eh*ODC such as it may also play a role similar to that of AZI in *E. histolytica*. Additionally, availability of *Eh*ODC crystal structure will be helpful in development of structure based anti-amoebiasis drugs.

### Accession number

Structure factors and final refined atomic coordinates for *Eh*ODC have been deposited in the Protein Data Bank (http://www.rcsb.org) with accession number 4AIB.
